# Aerobic Exercise Improves Signs of Restless Leg Syndrome in End Stage Renal Disease Patients Suffering Chronic Hemodialysis

**DOI:** 10.1155/2013/628142

**Published:** 2013-11-06

**Authors:** Mojgan Mortazavi, Babak Vahdatpour, Aida Ghasempour, Diana Taheri, Shahrzad Shahidi, Firouzeh Moeinzadeh, Bahareh Dolatkhah, Shahaboddin Dolatkhah

**Affiliations:** ^1^Isfahan Kidney Disease Research Center, Department of Nephrology, Isfahan University of Medical Sciences, Isfahan, Iran; ^2^Department of Physical Medicine and Rehabilitation, Isfahan University of Medical Sciences, Isfahan, Iran; ^3^Isfahan University of Medical Sciences, Isfahan, Iran; ^4^Isfahan Kidney Disease Research Center, Department of Pathology, Isfahan University of Medical Sciences, Isfahan, Iran; ^5^Department of Internal Medicine, Alzahra Hospital, Isfahan University of Medical Sciences, Isfahan, Iran; ^6^Department of Animal Science, Isfahan University of Technology, Isfahan, Iran

## Abstract

*Background*. Restless leg syndrome (RLS) is one of the prevalent complaints of patients with end stage renal diseases suffering chronic hemodialysis. Although there are some known pharmacological managements for this syndrome, the adverse effect of drugs causes a limitation for using them. In this randomized clinical trial we aimed to find a nonpharmacological way to improve signs of restless leg syndrome and patients' quality of life. *Material and Methods*. Twenty-six patients were included in the study and divided into 2 groups of control and exercise. The exercise group used aerobic exercise during their hemodialysis for 16 weeks. The quality of life and severity of restless leg syndrome were assessed at the first week of study and final week. Data were analyzed using SPSS software. *Results*. The difference of means of RLS signs at the first week of study and final week was −5.5 ± 4.96 in exercise group and −0.53 ± 2.3 in control group. There was not any statistical difference between control group and exercise group in quality of life at the first week of study and final week. *Conclusions*. We suggest using aerobic exercise for improving signs of restless leg syndrome, but no evidence was found for its efficacy on patient's quality of life.

## 1. Introduction

Restless leg syndrome (RLS) is one of the most common complaints among end stage renal disease (ESRD) patients [1]. This syndrome is characterized by a strong urge in the legs and other extremities which force the patient to move during rest [2]. Previous studies estimated the prevalence of RLS between ESRD patients to be 20% to 57% [3–5]. Multiparity, older age, sedentary life style, obesity, and positive family history are factors associated with RLS outbreak [6, 7]. In about 40% of patients presenting with RLS, a family history of this syndrome could be seen, and a dominant pattern of inheritance is known about its heritage [8]. RLS may occur idiopathic or secondary to conditions such as iron deficiency, pregnancy, peripheral neuropathy, rheumatoid arthritis, intake of psychopharmacological drugs, diabetes, or chronic kidney disease [9–13]. RLS has also substantial influence on quality of life of patients. Anxiety and depressed feelings are mental problems affecting patients with RLS that are specially seen in men [2]; however, neurological examination of patients suffering from RLS may be normal, but abnormalities on polysomnographic studies containing periodic limb movement might be seen in about 80% of patients [8, 14]. There are some diagnostic features which suggest RLS according to the international RLS study group (IRLSSG): (1) desire to move the extremities that is usually associated with paresthesias and/or dysesthesias, (2) motor restlessness, (3) worsening of symptoms particularly at rest, and (4) worsening of symptoms in the evening or night [15, 16]. 

Although there are not any known pathophysiological mechanisms to explain RLS, alleviation of symptoms with dopamine agonists (such as L-dopa) and worsening of symptoms with dopamine antagonists (such as metoclopramide) suggest that dopaminergic dysfunction in central nervous system may play an important role in the presence of symptoms [17, 18]. Secondary RLS in ESRD patients might be correlated with iron deficiency which has concurrence with anemia (due to lack of erythropoietin) [19] also in the absence of anemia in these patients [20, 21] and so iron treatment is an option to manage ESRD patients presenting RLS with anemia [22].

Although data suggesting effectiveness and safety of drugs for management of RLS are very limited [23]; the following drugs are assumed effective to decrease symptoms of RLS: levodopa, ropinirole, pramipexole, cabergoline, pergolide, and gabapentin [24, 25].

Lithium, selective serotonin reuptake inhibitors, and tricyclic antidepressant play a deteriorative role in RLS symptoms (related to their dopamine antagonists activity) [26].

Home short daily hemodialysis, as a nonpharmacological choice of therapy, is also associated with long-term improvement of RLS severity [27].

However, Aukerman et al. demonstrated the beneficial impact of exercise on the improvement of RLS severity and symptoms as a whole [28], but to the best of our knowledge there is only one published article on the effect of aerobic exercise on RLS symptoms between ESRD patients suffering chronic hemodialysis [29].

In this randomized clinical trial study we aimed to assess the effect of aerobic exercise on RLS severity and patients' quality of life during chronic hemodialysis.

## 2. Materials and Methods

Out of all patients undergone hemodialysis in Alzahra and Noor hospitals (two referral hospitals in Isfahan-center of Iran) with any etiology of renal failure, all patients were evaluated for RLS and according to inclusion criteria, 26 patients were enrolled in this randomized clinical trial. Inclusion criteria were hemodialysis for at least 3 months, sufficient dialysis for at least 3 times weekly, presence of RLS, ferritin (Fr) >100 ng/mL, and transferrin saturation rate (TSAT) >20%. Patients with the following disorders were excluded: musculoskeletal disorders which incapacitated them from physical activity, history of ischemic heart disease (recent myocardial infarction or unstable angina), any catabolic process such as malignancies, opportunistic infections, and infections needing antibiotic therapy during the last 3 months.

All of the participants (26 patients) were informed about the details of the experiment and then were randomly divided into control group (13 patients) and exercise group (13 patients). Patients in the exercise group pedaled the bicycle (Medi-bike made in Switzerland) 3 times a week for 16 weeks. Each time for exercise consisted of 30 minutes of continuous pedaling between hour 2 and 3 during dialysis. For prevention of cardiovascular disorders, the first 5 minutes was spent for warm-up by slow heartbeat which made the body ready for main exercise. After that the main exercise started and participants pedaled for 20 minutes, and finally 5 minutes of cool-down was performed to lower heartbeat. Exercise intensity was evaluated using Borg scale [30] and Borg scale of 10–12 applied to it.

Patients' blood pressure and heart rate were determined before and after dialysis and at hour 2 and 3 during dialysis. Systolic blood pressure more than 160 mmHg or diastolic blood pressure more than 110, chest pain, dyspnea, body temperature more than 37.8°C, cardiac arrhythmias, signs of insufficient tissue perfusion, and neurological signs such as vertigo or imbalance were criteria for interrupting exercise. These complications did not occur to any of the participants during the test.

Severity of RLS was evaluated using RLSQ questionnaire [31] at the beginning of the study and week 16. Quality of life was evaluated by using SF-63 [32] questionnaire at the beginning of the study and week 16. Finally, gathered data analyzed by SPSS-16 software (Chicago, IL, USA), dependent *t*-test, and independent *t*-test were used for comparing intragroup and intergroup data. 

## 3. Results

Out of all patients, 18 patients (69%) were males and 8 patients (31%) were females. Mean age of patients was 41.5 ± 12.1 as a whole, 32.3 ± 6.7 in exercise group and 47.1 ± 13.1 in control group.

Considering Table 1, scores of restless leg syndrome showed a descending pattern in exercise group during weeks of experiment, but no significant difference in control group was seen.

Difference of means between the first week of experiment and the final week was −5.5 ± 4.96 in exercise group and −0.53 ± 2.3 in control group. Comparing groups by using independent *t*-test showed significant statistical difference between them (*P* value = 0.003). 

Assessment of quality of life indicated mean number of 118 ± 8.4 in control group and 141 ± 5.7 in exercise group at the first week of study. The final assessment of quality of life at the end of study showed mean number of 116 ± 8.32 in control group and 142 ± 6.1 in exercise group. The independent *t*-test did not show any statistical difference between groups (*P* value = 0.61). 

## 4. Discussion

The goal of this study was to find a suitable non-pharmacological interaction for decreasing the severity of restless leg syndrome's signs in patients suffering chronic hemodialysis. However, prevalence of this syndrome is not rare in hemodialysis patients, but only 26 patients met our criteria to participate in the study and this issue was assumed as a limitation in our study. By our data, signs of restless leg syndrome significantly improved during aerobic exercise. Sakkas et al. had also suggested aerobic exercise as an appropriate management for restless leg syndrome in patients suffering chronic hemodialysis [29]. Although Sakkas et al. found an improving pattern for quality of life during weeks of exercise in patients with RLS [29], we did not find a significant impact of aerobic exercise on quality of life between patients with RLS using aerobic exercise and those in control group; perhaps with prolongation of study, the quality of life may improve. This discrepancy of findings may also come from other aspects of life influencing quality of life between our patients and patients in previous studies. Because of adverse effects of RLS on patients' life, it is really important to find suitable ways for alleviating their symptoms and improving quality of life between them. Future studies with more patients could clarify whether aerobic exercise has an improving effect on patients' quality of life.

We suggest doing aerobic exercise for improving signs of restless leg syndrome for patients suffering chronic hemodialysis, but more studies are recommended for evaluating the role of aerobic exercise to improve quality of life.

## Figures and Tables

**Table 1:** Mean of restless leg syndrome scoring in both groups.

Group	Timing	
	Start of the experiment	At the end of experiment
Exercise	19.3 ± 3.8	13.9 ± 5.9
Control	22.8 ± 5.5	22.3 ± 4.6
